# Are COVID-19 mRNA vaccine side effects severe enough to cause missed work? Cross-sectional study of health care-associated workers

**DOI:** 10.1097/MD.0000000000028839

**Published:** 2022-02-18

**Authors:** David A. Cohen, Patricia Greenberg, Brielle Formanowski, Payal D. Parikh

**Affiliations:** a Department of Medicine, Rutgers Robert Wood Johnson Medical School, New Brunswick, NJ; b Biostatistics and Epidemiology Services Center, Rutgers School of Public Health, Rutgers University, Piscataway, NJ; c Department of Biostatistics and Epidemiology, Rutgers School of Public Health, Rutgers University, Piscataway, NJ.

**Keywords:** COVID-19, missed work, Moderna, Pfizer-BionTech, side effects, vaccination

## Abstract

The Coronavirus Disease 2019 (COVID-19) pandemic, caused by the severe acute respiratory syndrome-coronavirus-2, has claimed 5,587,549 lives worldwide as of January 20, 2022. Fortunately, large-scale vaccination can mitigate the impact of COVID-19 by making the disease milder and less common. Although 75.2% of the United States population has received at least 1 dose of a COVID-19 vaccines thus far, concerns regarding vaccine side effects have contributed to vaccine hesitancy. Furthermore, nearly 50% of adults in the United States are concerned not only about side effects, but about their downstream impact, including missed work.

The goal of this cross-sectional study was to investigate the effect of messenger RNA vaccine adverse effects on the propensity to miss work among employees associated with a single, large academic health center.

Using Qualtrics, all employees, including faculty, staff, and trainees, of 5 large departments were surveyed to determine whether they received the COVID-19 vaccine and which type, and any symptoms they experienced after receipt of either vaccine dose. We hypothesized that vaccine recipients would be more likely to miss work or feel sick enough to miss work following the second dose.

Thirty-seven percent of respondents experienced events severe enough that they needed to miss work from either of the doses, with the majority (27.8%) related to the second dose. These findings are consistent with and expand on the results from the phase 3 trials for Pfizer-BionTech and Moderna, which showed that vaccine side effects were more common after the second dose than after the first dose. Our statistically significant finding was more common among Asians, women, trainees/house staff, and nonphysician clinical employees.

With an increasing number of individuals taking the vaccine, employers will need to account for the impact of adverse effects on their employees’ ability to work. These findings will further help organizations better plan for staffing as vaccinations increase to mitigate the spread of COVID-19.

## 1 Introduction

The Coronavirus Disease 2019 (COVID-19) pandemic, caused by the severe acute respiratory syndrome-coronavirus-2, has claimed 5,587,549 lives worldwide as of January 20, 2022.^[1]^ There are limited effective therapeutic options with emerging variants that are even more transmissible than the original severe acute respiratory syndrome-coronavirus-2. Additionally, scientific models suggest that the virus will continue to circulate throughout the global population indefinitely.^[2]^ Fortunately, large-scale vaccination can mitigate the impact of COVID-19 by making the disease more mild and less common.

As of January 20, 2022, 75.2% of the total population had received at least 1 dose of a COVID-19 vaccine that received emergency use authorization by the Food and Drug Administration, whereas and 63.1% were considered fully vaccinated.^[3]^ Mild side effects such as injection site reactions, fatigue, headache, and low-grade fever are common with both vaccines.^[^4^,^^5^^]^ The Centers for Disease Control and Prevention reported that anaphylaxis, the most severe side effect reported in the messenger RNA (mRNA) vaccines, occurred in 11.1 and 2.5 cases per million doses of the Pfizer-BionTech and Moderna vaccines, respectively.^[^6^,^^7^^]^ Although the 2 studies that led to emergency use authorization did not report any episodes of anaphylaxis, less than 2% of the participants who received either vaccine reported severe or life-threatening systemic adverse events. Conversely, moderate side effects (Grade 2 or higher) occurred in 33% of participants who received the Pfizer vaccine, whereas almost 21% of participants who received the Moderna vaccine experienced a related adverse event.^[^4^,^^5^^]^ In both studies, when side effects occurred, they were more common following the second dose as compared to the first dose.

Many individuals delay or refuse to take the vaccine due to concerns about the severity of side effects, including almost 20% of US health care workers who are not yet vaccinated, according to a recent survey.^[8]^ Given that everyone 12 years of age and older is eligible to receive a vaccine, both companies that created the mRNA vaccines are performing trials in younger children, and the White House Administration has required the vaccine of many Americans,^[9]^ the coming months will continue to see significantly increasing numbers of vaccinated people, particularly among the working-age population. A recent Kaiser Family Foundation poll identified that nearly 50% of adults in the United States are concerned not only about side effects themselves, but also about missing work due to the side effects of the vaccine.^[8]^ However, no study to date has evaluated whether the adverse events associated with either the Pfizer-BionTech or Moderna vaccines led to participants missing days of work.

The goal of this study was to investigate the relationship between mRNA vaccine adverse effects and the propensity for missing work on account of the symptoms among employees associated with a single, large academic health center. Furthermore, we examined whether the severity of side effects from the first dose was predictive of the severity of the second dose, and whether this would correlate with missed work. We hypothesized that there would be a small frequency of work absenteeism following the first dose, but that this would increase significantly following the second dose. Accordingly, we hypothesized that report of symptom severity would correlate with the need to miss work.

## 2 Methods

This cross-sectional study obtained data via a Qualtrics survey that was sent out via email to all employees in the departments of Family Medicine, Internal Medicine, Obstetrics and Gynecology, Pediatrics, and Surgery at Robert Wood Johnson Medical School in January and February 2021. These departments were chosen because they have the highest number of faculty and would subsequently yield the most participants. The employees surveyed included residents and fellows in training; paid staff; and paid and volunteer faculty members. To limit recall bias, the survey was emailed to employees during the first release of the vaccines and closed 4 weeks after it was first sent out. The survey included 30 related to the respondent's role in the hospital, history of medical comorbidities associated with worse COVID-19 outcomes, regularity of influenza vaccination, and other general demographics (i.e. age, gender, race, and ethnicity). Additional questions included whether participants had received the COVID-19 vaccine, which vaccine they received, and symptoms they had experienced after they received 1 or both doses of the vaccine. Severity of symptoms experienced after receipt of the vaccine (side effects) was grouped into 4 categories: none, mild, moderate, and severe (Table 1). The main outcome of this study was whether the respondent needed to miss work because of the side effects of their first vaccination and/or from their second vaccination, if applicable.

**Table 1:** Side effect severity from vaccine.

No symptoms at all
Minimal severity: minimal localized pain, mild headache, fatigue, nausea, chills, palpitations, runny nose, sore throat, decreased appetite
Moderate severity: moderate localized pain, fever, vomiting, diarrhea, severe headache, hives
Severe severity: severe localized pain, allergic reaction, hospitalization

General unadjusted results were summarized using frequency and percentage and compared using Pearson Chi-Square or Fisher Exact tests for categorical measures and Wilcoxon Rank Sum tests for ordinal measures. McNemar and Wilcoxon Signed Rank tests were used for unadjusted results comparing participant reported outcomes for dose 1 vs dose 2 for the same respondents. Multivariable logistic regression, with stepwise variable selection, was used to test the association of dose 2 symptom severity with the need to stay home after dose 2, adjusting for age group, gender, race, ethnicity, role, comorbidity status, flu shot status, symptom severity after dose 1, and whether they missed work after dose 1. All analyses were performed using Statistical Analysis System version 9.4 (Statistical Analysis System Institute Inc., Cary, NC) and all 2-sided *P* values <.05 were considered statistically significant. The study was approved by the Institutional Review Board at Rutgers Robert Wood Johnson Medical School.

## 3 Results

A total of 861 survey responses were received, for a response rate of 50%. Among those, 192 (22.3%) respondents provided information about their vaccine-related outcomes after having received only 1 dose and 529 (61.4%) healthcare workers provided vaccine-related outcomes after receiving both vaccination doses, at the time of their survey response. All other respondents had either not received any vaccination doses at the time of survey response or opted not to provide information about their vaccine-related outcomes. Of the 529 respondents, 8.2% were 30 years old or younger, 27.2% were 31 to 45 years old, 31.9% were 46 to 60 years old, and 32.7% were over 60 years old. Approximately 70.3% of the respondents were attending physicians, with trainees/house staff making up 11.7%, nurses accounting for 8.7%, and other more specific clinical and nonclinical roles making up the remaining 9.3%. Females accounted for 54.2% of the sample, 68.4% self-reported as White, 30.1% reported having medical comorbidities that are related to worse COVID-19 outcomes, and 94.1% reported receiving the flu shot every year (Table 2).

**Table 2:** Baseline characteristics.

Demographic measure	Total study sample (861)	Group 1: vaccinated (821)	Group 2: unvaccinated (40)
Age, n (%)			
30 or younger	60 (7.0)	57 (6.9)	3 (7.5)
31-45	254 (29.5)	232 (28.3)	22 (55.0)
46-60	270 (31.4)	262 (31.9)	8 (20.0)
61 and older	271 (31.4)	265 (32.3)	6 (15.0)
Not reported	6 (0.7)	5 (0.6)	1 (2.5)
Race, n (%)			
Asian	212 (24.6)	209 (25.5)	3 (7.5)
Black	67 (7.8)	51 (6.2)	16 (40.0)
White	565 (65.6)	546 (66.5)	19 (47.5)
Other	5 (0.6)	5 (0.6)	0 (0.0)
Not reported	12 (1.4)	10 (1.2)	2 (5.0)
Hispanic/Latino/Spanish Origin, n (%)			
Yes	55 (6.4)	49 (6.0)	6 (15.0)
No	797 (92.6)	765 (93.2)	32 (80.0)
Not reported	9 (1.0)	7 (0.8)	2 (5.0)
Gender, n (%)			
Female	505 (58.7)	471 (57.3)	34 (85.0)
Male	349 (40.5)	344 (41.9)	5 (12.5)
Non-binary/third gender/other	3 (0.3)	3 (0.4)	0 (0.0)
Not reported	4 (0.5)	3 (0.4)	1 (2.5)
Department, n (%)			
Family medicine	34 (3.9)	32 (3.9)	2 (5.0)
Internal medicine/department of medicine	427 (49.6)	419 (51.0)	8 (20.0)
Obstetrics & gynecology	71 (8.3)	65 (7.9)	6 (15.0)
Pediatrics	244 (28.3)	231 (28.2)	13 (32.5)
Surgery/plastic surgery	66 (7.7)	60 (7.3)	6 (15.0)
Other	19 (2.2)	14 (1.7)	5 (12.5)
Flu shot status, n (%)			
Every year	766 (89.0)	753 (91.7)	13 (32.5)
Most/some years	78 (9.0)	62 (7.6)	16 (40.0)
Never	17 (2.0)	6 (0.7)	11 (27.5)
Medical comorbidities associated with Covid-19, n (%)			
Yes	267 (31.0)	250 (30.4)	17 (42.5)
No	575 (66.8)	558 (68.0)	17 (42.5)
Not reported	19 (2.2)	13 (1.6)	6 (15.0)
Role, n (%)			
Non-clinical	111 (12.9)	92 (11.2)	19 (47.5)
Nurse	70 (8.1)	66 (8.0)	4 (10.0)
Physician	568 (66.0)	555 (67.6)	13 (32.5)
Trainee	80 (9.3)	80 (9.8)	0 (0.0)
Other clinical	14 (1.6)	14 (1.7)	0 (0.0)
Other (not classifiable)	18 (2.1)	14 (1.7)	4 (10.0)

Overall, among those who had received both doses (n = 529), 333 (63.0%) did not report needing to miss work after either dose, whereas 16 (3.0%) needed to miss work after dose 1 but not dose 2, 147 (27.8%) needed to miss work after dose 2 but not dose 1, and 33 (6.2%) needed to miss work after both doses (*P* < .001, Table 3). In an examination of differences between vaccine manufacturers (Pfizer vs Moderna), there was no significant difference in the surveyed employees’ need to miss work after they received the first dose. However, there was a significant difference in the number of those who reported needing to miss work after the second dose, where 49.4% of those who received Moderna reported needing to miss work, and only 26.2% of those who received Pfizer reported needing to miss work (*P* < .001, Table 4).

**Table 3:** Overall needing to miss work.

		Needed to miss work after second dose	
Total n = 529		No	Yes
Needed to miss work after first dose	No	333 (63.0%)	147 (27.8%)
	Yes	16 (3.0%)	33 (6.2%)

**Table 4:** Needing to miss work by vaccine manufacturer.

	Manufacturer: Pfizer (n = 351)^∗^		*P* value
Needed to miss work due to vaccination side effects, n (%)	First dose	Second dose	*P* < .01
No	323 (92.0)	259 (73.8)	
Yes	28 (8.0)	92 (26.2)	

	Manufacturer: Moderna (n = 178)^∗^		
Needed to miss work due to vaccination side effects, n (%)	First dose	Second dose	*P* < .01
No	157 (88.2)	90 (50.6)	
Yes	21 (11.8)	88 (49.4)	

∗ 14 respondents did not indicate which type of vaccine they had received.

In terms of vaccination symptom severity (“None”, “Minimal”, “Moderate”. “Severe”), among those who had received both doses (n = 529), 321 (60.7%) reported having the same level of symptom severity for both doses, whereas 132 (25.0%) and 76 (14.3%) reported having more severe and less severe symptoms for dose 2 vs dose 1, respectively (*P* < .001). Most notably, 12 (17.4%) of the respondents who reported no symptoms after dose 1, reported moderate symptoms after dose 2, and 78 (22.6%) of the respondents who reported minimal symptoms after dose 1, reported moderate or severe symptoms after dose 2. No respondents reported severe symptoms after either dose (Table 5).

**Table 5:** Overall symptom severity.

Measure or outcome	First dose (n = 529)	Second dose (n = 529)	*P* value
Symptom severity, n (%)			*P* < .01
None (0)	69 (13.0)	36 (6.8)	
Minimal (1)	345 (65.2)	342 (64.7)	
Moderate (2)	106 (20.0)	146 (27.6)	
Severe (3)	9 (1.7)	5 (1.0)	

After applying stepwise variable selection, the remaining predictors of whether healthcare workers would need to stay home after the second vaccine dose included race, gender, hospital role, whether they needed to miss work after the first dose, and second dose symptom severity. Compared to those who had severe symptoms after the second dose, there was no significant difference for those who reported minimal (*P* = .09) and moderate (*P* = .57) symptoms; however, for those who reported no symptoms after their second dose, there was a significant decrease in the likelihood of needing to stay home after dose 2 (OR = 0.01 [95% CI: 0.00, 0.28], *P* = .01). For those who did not report needing to miss work after the first dose, there was a significant decrease in needing to miss work after the second dose as well (0.19 [0.09, 0.39], *P* < .001). Additionally, compared to those who self-reported as Black, there was no significant difference for those who self-reported as White (1.21 [0.42, 3.52], *P* = .72) or “Other” (1.82 [0.09, 36.2], *P* = .69), with a trending significant increase for those who self-reported as Asian (2.97 [0.98, 8.96], *P* = .05). Compared to males, females showed a trending significant increase (1.61 [1.0, 2.58], *P* = .05), and compared to faculty, both trainees/house staff (1.97 [1.06, 3.67], *P* = .03) and other specific clinical roles (6.88 [1.55, 30.48], *P* = .01) showed a significant increase in the need to stay home after dose 2 (Table 6).

**Table 6:** Logistic regression model for needing to miss work.

Predictor	β coefficient (SE)	Odds ratio with 95% CI	*P* value
Intercept	−2.85 (1.18)	–	.02
Race = Asian	1.09 (0.56)	2.97 (0.98, 8.96)	.05
Race = other	0.60 (1.52)	1.82 (0.09, 36.18)	.69
Race = White	0.19 (0.54)	1.21 (0.42, 3.52)	.72
Gender = female	0.47 (0.24)	1.61 (1.00, 2.58)	.05
Role = non-clinical	0.82 (0.44)	2.26 (0.96, 5.30)	.06
Role = nurse	0.71 (0.41)	2.03 (0.91, 4.50)	.08
Role = other (non-classifiable)	−0.38 (0.98)	0.69 (0.10, 4.65)	.70
Role = other clinical	1.93 (0.76)	6.88 (1.55, 30.48)	.01
Role = trainee	0.68 (0.32)	1.97 (1.06, 3.67)	.03
Missed work after first shot = no	−1.66 (0.37)	0.19 (0.09, 0.39)	<.001
Symptom severity (second dose) = minimal	2.34 (1.04)	10.39 (1.36, 79.58)	.02
Symptom severity (second dose) = moderate	3.67 (1.05)	39.36 (5.04, 307.49)	.001
Symptom severity (second dose) = severe	4.34 (1.56)	76.76 (3.61, Inf)	.01

CI = confidence interval, SE = standard error.

## 4 Discussion

In this survey-based study of paid and volunteer faculty, clinical and nonclinical trainees, and staff affiliated with a large academic health center, adverse events from the mRNA COVID-19 vaccines (Pfizer-BionTech and Moderna) were severe enough to interfere with work. Our results showed that 37% of participants experienced events severe enough that they needed to miss work from either of the doses, with the majority (27.8%) related to the second dose effects. As predicted in our hypothesis, vaccine recipients were significantly more likely to miss work or feel sick enough to miss work following the second dose (34% of respondents) than after the first dose (9.2% of respondents). These findings are consistent with results from the phase 3 trials for Pfizer-BionTech and Moderna, which showed that vaccine side effects were more common after the second dose than after the first dose.

Interestingly, additional analysis showed that there was no significant difference in respondents missing work after the first dose of either vaccine but there was a significant difference in respondents missing work or feeling unwell enough to want to miss work after the second dose with both the Pfizer and Moderna vaccines. Additionally, Moderna was associated with a nearly 2-fold increase in second-dose adverse events causing respondents to miss work as compared to Pfizer. This finding is consistent with prior evidence that adverse effects are more frequent in patients who receive the Moderna vaccine than in those receiving the Pfizer vaccine.^[10]^ However, our study was the first to look at this solely through the lens of its effect on workplace attendance and the first to analyze a statistically significant difference.

When applying stepwise regression for demographics, we discovered that females (when compared with males), trainees/house staff (when compared with faculty), and non-attending/non-nursing roles (when compared with faculty) were more likely to need to stay home after dose 2. In terms of gender, this finding is consistent with prior research that has shown that females report more adverse effects from vaccines that do males.^[11]^ However, this is the first study to look at these rates following the COVID-19 vaccine, or to look at these rates comparing different medical personnel following any vaccine. In terms of race and ethnicity, our study found that there was no statistical difference identified in the outcomes related to missing work due to vaccine side effects in self-reported Black and self-reported White participants. A noted positive correlation was noted in self-reported Asians. Here we show that while more than half (55%) of unvaccinated Black adults and 64% of unvaccinated Hispanic adults are concerned about having to miss work,^[8]^ the adverse events reported by either group did not lead to missed work in our population

Our study had several limitations. First, we surveyed individuals associated with a single institution in New Jersey. Therefore, the results may not be generalizable to other locations in the country or to individuals outside of the medical field. Because our survey collected data at the beginning of 2021 when the Pfizer-BionTech and Moderna vaccines were the only available options, the results do not provide any information about adverse events from other vaccines against COVID-19, nor does it tell us whether the respondents have similar symptoms after receiving other vaccines. Furthermore, we did not collect information about which specific adverse events interfered with respondents’ work attendance. Similarly, because the patients determined the severity of the side effects, there is clear reporting bias involved. However, given that our study examined the symptoms’ impact on participants ability or willingness to work, the participants’ perceived severity is more relevant than what the specific event was. Furthermore, while certain demographics were associated with a need to stay home after vaccine receipt, our survey did not investigate the reasons for these differences. Our study is not generalizable to individuals who receive other COVID-19 vaccines, such as that produced by Johnson & Johnson/Janssen. Furthermore, the results from our study should not be applied to the Pfizer-BionTech and Moderna “booster shots” recently approved.

In our literature search, no other studies have looked at the need to stay home after receipt of the mRNA COVID-19 vaccines. Therefore, our study is unique and has significant implications for the public. With an increasing number of individuals taking the vaccine, employers must consider the impact of adverse effects on their employees’ ability to work. Depending on the type of work, employers can consider either staggering vaccination days for the employees (to minimize missed workers) or vaccinate all employees on the same day and give the next day as a planned day off from work. Additionally, with school-aged persons increasingly able to take the vaccines, the effects on school attendance and learning must be taken into consideration. Finally, with the possibility that booster shots may be needed, similar studies are warranted to explore the effect of a third vaccine dose on the workplace.

Further research should be directed toward understanding which adverse events are most likely to interfere with recipients’ work attendance. If this information is known, interventions may be explored to prevent such events. Likewise, a greater understanding of why there are racial, gender, and professional differences, may help minimize the effect of vaccine adverse events. Given that our study only surveyed adults, similar studies of adolescents and children should be conducted to understand the potential impact on school attendance. With the increasing use of the Pfizer-BionTech and Moderna “booster shots”, similar studies should also be performed to investigate the effect of these additional vaccinations on work interference.

## 5 Conclusions

Over one-third of recipients of either of the 2 COVID-19 mRNA vaccines (Pfizer-BionTech and Moderna) experienced adverse events that were severe enough to cause them to either miss work or wish they would have. This finding was more common among Asians, women, trainees/house staff, and nonphysician clinical employees. Further research is needed to understand the drivers of these findings and how to mitigate such events.

## Author contributions

**Conceptualization:** David A Cohen, Patricia Greenberg, Payal Parikh.

**Data curation:** David A Cohen, Patricia Greenberg, Brielle Formanowski.

**Formal analysis:** Patricia Greenberg, Brielle Formanowski.

**Investigation:** David A Cohen, Patricia Greenberg, Brielle Formanowski, Payal Parikh.

**Methodology:** David A Cohen, Patricia Greenberg, Brielle Formanowski.

**Project administration:** David A Cohen.

**Resources:** David A Cohen.

**Software:** Patricia Greenberg, Brielle Formanowski.

**Supervision:** David A Cohen, Patricia Greenberg.

**Validation:** Patricia Greenberg.

**Visualization:** David A Cohen, Payal Parikh.

**Writing – original draft:** David A Cohen.

**Writing – review & editing:** David A Cohen, Patricia Greenberg, Brielle Formanowski, Payal Parikh.
